# A quantitative PCR assay for the detection and quantification of *Septoria pistaciarum*, the causal agent of pistachio leaf spot in Italy

**DOI:** 10.1371/journal.pone.0286130

**Published:** 2023-05-19

**Authors:** Mounira Inas Drais, Giorgio Gusella, Angelo Mazzaglia, Giancarlo Polizzi

**Affiliations:** 1 Dipartimento di Scienze Agrarie e Forestali, Università degli Studi della Tuscia, Viterbo, Italy; 2 Dipartimento di Agricoltura, Alimentazione e Ambiente, Sezione di Patologia Vegetale, University of Catania, Catania, Italy; International Institute of Tropical Agriculture, KENYA

## Abstract

Septoria leaf spot is one of the most widespread diseases affecting pistachio (*Pistacia vera*) in countries of the Mediterranean region. *Septoria pistaciarum* was recently confirmed as the causal agent of this disease in Italy. Currently, the detection of *S*. *pistaciarum* relies on isolation techniques. These require significant amounts of labor, and time for completion. Also, a reliable identification requires the sequencing of at least two housekeeping genes, in addition to the morphological observations. To accurately detect the presence and quantify *S*. *pistaciarum* in pistachio tissues, a molecular tool was necessary. We designed applicable primers that allow reliable amplification of the β-tubulin gene. The amplification of target DNA was highly efficient, with a 100% success rate, and the assay was able to detect as little as 100 fg/rxn of pure fungal DNA. When tested in artificial mixtures of plant and pathogen DNAs, the assay was able to detect the pathogen consistently at a limit of detection of 1 pg/rxn. The assay was also effective in identifying the pathogen in naturally infected samples, providing rapid detection in all symptomatic specimens. The resulting qPCR assay is an improved detection tool for accurate diagnosis of *S*. *pistaciarum* that can also contribute to better understand the population dynamics of the pathogen in the orchard.

## Introduction

Pistachio (*Pistacia vera*) is an important Mediterranean crop. Iran, United States, China, and Turkey are considered the world’s top producers (https://www.fao.org). Italian pistachio production is concentrated in the southern regions, of which Sicily is considered the main production area of the national territory. In Sicily pistachio fruit of the traditional area of Bronte, due to their peculiar organoleptic characteristics are protected by the label of PDO (protected designation of origin) “Green Pistachio of Bronte”, as well as pistachios from Agrigento and Caltanissetta provinces are also protected by the PDO label of “Pistacchio di Raffadali”.

Among the fungal diseases affecting pistachio, Verticillium wilt (*Verticillium dahliae*), Botryosphaeria panicle and shoot blight (*Botryosphaeriaceae* spp.), and Alternaria late blight (*Alternaria* spp.) are considered the main limiting factors for the cultivation [1–3]. Recently surveys for fungal diseases were conducted in Sicily to update the knowledge on relevant diseases spread in the main cultivated area. Botryosphaeria panicle and shoot blight has been detected in the new orchards of Agrigento and Caltanissetta provinces, and related to three species (i.e., *Botryosphaeria dothidea*, *Neofusicoccum hellenicum* and *N*. *mediterraneum*) with *N*. *mediterraneum* being the most spread among the orchards [4]. Among canker and dieback diseases of pistachio, other fungal pathogens have been reported and characterized during these years such as *Cytospora pistaciae*, *Eutypa lata*, and *Leptosillia pistaciae* (ex *Liberomyces pistaciae*), considered in the traditional area of Bronte one of the major canker pathogens of this crop [5–7]. Other minor diseases have been described, in particular, fruit blight caused by *Arthrinium xenocordella* and pistachio fruit rust caused by *Tuberculina persicina* [8, 9].

Regarding leaf diseases, Septoria leaf spot is one of the most widespread pistachio diseases worldwide and, especially in important productive areas, severe losses are expected if this occurs [10]. In Spain in fact, high level of defoliation was observed especially in those fields not treated with fungicides [11]. In Sicily, infections occur every year in the field, but constant observations showed that in absence of preventive fungicide treatments, before the appearance of early symptoms, severe defoliation up to 100% can occur. Three different *Septoria* spp. have traditionally been associated with pistachio, i.e., *S*. *pistaciae*, *S*. *pistaciarum* and *S*. *pistacina*. Desmazieres in 1842 [12] in France described *S*. *pistaciae* causing leaf spots on *P*. *vera*. In 1901 Allescher [13] introduced *S*. *pistacina*, and some years later, Caracciolo in 1934 [14] reported a third species named *S*. *pistaciarum* in Sicily. Recently, Crous *et al*. in 2013 [15] elucidated the taxonomy of *Septoria*-like pathogens associated with pistachio, distinguishing *Cylindroseptoria pistaciae*, *Pseudocercospora pistacina* (ex *S*. *pistacina*), *S*. *pistaciae* (being part of *S*. *protearum* species complex) and *S*. *pistaciarum* (*S*. *pistaciae* and *S*. *pistaciarum* being part of *Septoria s*. *str*.).

Based on these advances, in 2021 Gusella *et al*. [16], investigated the Septoria leaf spot disease in Sicily and confirmed that the isolates recently collected accommodate within the clade of *S*. *pistaciarum*. *Septoria pistaciarum* was also reported in Arizona, New Mexico (US), Greece, India, Spain, and in East-Mediterranean and Southeast Anatolian regions [1, 17–20]. Disease symptoms appear from springtime to the end of summertime as irregular red lesions with black margins on both sides of the leaves, usually confined by leaf veins, and coalescing with time [16]. Presence of leaf lesions affects the performance of the plants in terms of photosynthesis, as demonstrated, for example, for blueberry of which the assimilation of CO_2_ is compromised by the infections by *S*. *albopunctata* [21]. Severe inoculum pressure and favorable environmental conditions lead to premature defoliation of pistachio trees, affecting the current bearing shoots and the physiological processes of carbohydrates assimilation required for bud differentiation.

Regarding management of this pathogen, fungicides (QoI and SDHI) are the only available tools to control pistachio leaf spot. To limit the number of field sprays, detection of latent infections within the tissues could represent an important point for a sustainable use of pesticide through valuable and accurate spray program and for monitoring the curative effects of the fungicides during the incubation period of fungal infections. Moreover, different aspects of *S*. *pistaciarum* life cycle, biology, and disease epidemics are still unclear. All these issues in epidemiological and biological research need a proper methodology. Sometimes traditional methods are inadequate or slower in obtaining quantitative results, reason why a well-developed quantitative real-time PCR (qPCR) methodology could represent a key point for an accurate diagnosis and understanding of epidemiological and biological phenomena. Recently, several studies have focused on developing qPCR detection techniques for nut trees fungal pathogens like *Gnomoniopsis castaneae* in chestnut tissues and *Piggotia coryli* in hazelnut tissues [22, 23]. Information derived from the qPCR in terms of inoculum density could be combined with weather or micro-environmental data in the field, in order to estimate infection rate, trends in disease development and risk of disease development, as proposed in California for canker pathogens [24]. Until now, no qPCR methodology was assessed for *S*. *pistaciarum*. On the basis of all the great potentials of this methodology for epidemiology, biology and disease management of pistachio leaf spot disease, the aim of our study was to assess and validate a quantitative PCR assay for the detection and quantification of *S*. *pistaciarum*.

## Material and methods

### Fungal strains and culture conditions

Fungal isolates utilized in the present study (Table 1) were sourced from the culture collection of the Dipartimento di Agricoltura, Alimentazione e Ambiente of the University of Catania. Among them, isolates of *S*. *pistaciarum* S1 (CBS 146142), S13 (CBS 146141), SE and S14 as well as the isolate of *N*. *mediterraneum* P107 were previously characterized [4, 16]. All the other isolates were identified according to their morphological and cultural characteristics. For the isolation, small pieces of 0.2–0.3 cm^2^ were surface sterilized for 1 min in 1.5% sodium hypochlorite solution, rinsed with sterile water, air dried in a laminar hood, and placed on Potato Dextrose Agar (PDA, Lickson, Vicari, Italy) amended with 100 mg/liter of streptomycin sulfate (Sigma-Aldrich, St. Louis, MO, USA) (APDA). All the Petri plates were incubated at 25°C for three to ten days. The isolation frequency was calculated according to the formula:

$$F=\frac{N_{Sep}}{N_{Tot}}\times 100$$

where F is the frequency of *S*. *pistaciarum*; N_Sep_ is the number of leaf fragments from which *S*. *pistaciarum* was isolated; and N_Tot_ is the total number of leaf fragments from which fungal isolation was conducted.

**Table 1:** List of fungi and plants.

Genus and species	Isolate code	Tissue	Host	qPCR result
*Alternaria* sp.	E11	Bud	*Pistacia vera*	-
*Alternaria* sp.	E13	Fruit epicarp	*Pistacia vera*	-
*Alternaria* sp.	E16	Leaf	*Pistacia vera*	-
*Alternaria* sp.	E19	Fruit epicarp	*Pistacia vera*	-
*Botrytis* sp.	PB3	Bud	*Pistacia vera*	-
*Chaetomium* sp.	PB7	Bud	*Pistacia vera*	-
*Cladosporium* sp.	E9	Fruit epicarp	*Pistacia vera*	-
*Colletotrichum* sp.	E5	Leaf	*Pistacia vera*	-
*Diaporthe* sp.	PB13	Bud	*Pistacia vera*	-
*Neofusicoccum mediterraneum*	P107	Fruit epicarp	*Pistacia vera*	-
*Neurospora* sp.	E1	Fruit epicarp	*Pistacia vera*	-
*Neurospora* sp.	E3	Bud	*Pistacia vera*	-
*-*	P0	Leaf	*Pistacia vera*	-
*Septoria pistaciarum*	SE	Leaf	*Pistacia vera*	+
*Septoria pistaciarum*	S14	Leaf	*Pistacia vera*	+
*Septoria pistaciarum*	S1	Leaf	*Pistacia vera*	+
*Septoria pistaciarum*	S13	Leaf	*Pistacia vera*	+
*Septoria pistaciarum*	S4	Leaf	*Pistacia vera*	+
*Septoria pistaciarum*	SL	Leaf	*Pistacia vera*	+
*Septoria pistaciarum*	SD5	Leaf	*Pistacia vera*	+
*Septoria pistaciarum*	SD2	Leaf	*Pistacia vera*	+
*Septoria pistaciarum*	S7	Leaf	*Pistacia vera*	+
*Septoria* sp.	SL10+	Fruit spot	*Citrus limon*	-
*Trichoderma* sp.	E10	Bud	*Pistacia vera*	-

List of fungi and plants used for testing the analytical specificity of a real-time qPCR assay targeting *Septoria pistaciarum* DNA, and the corresponding results.

Fungal colonies emerging from isolations were visually inspected and purified by transferring a tip of actively growing mycelium into another plate. The resulting single spore/ hyphal-tip isolates were stored at 4°C.

### DNA extraction

Total genomic DNA was extracted from 10-day-old pure fungal cultures by scraping the mycelium with a sterile scalpel and following the NucleoSpin® Plant II kit manufacturer’s protocol (MACHEREY-NAGEL, Düren, Germany). The same protocol was used to extract plant DNA. DNA concentration was measured with Qubit (Thermo Fisher,Waltham, MA) using the High Sensitivity dsDNA Assay kit. Extracted DNAs were stored at −20°C until further analysis.

### Quantitative PCR detection method

Preliminary screening was carried out to select the most informative DNA regions for *S*. *pistaciarum* in fungal housekeeping genes (i.e., ITS rDNA, partial β-tubulin, nuclear ribosomal RNA gene large subunit (LSU) and translation elongation factor 1 alpha EF1α). Available sequences of *S*. *pistaciarum* and additional closely related species were downloaded from the NCBI database and aligned using CLUSTALW (multiple sequence alignment) in MEGA 10 software [25] looking for regions suitable for specific primers design. Primer3 [26] was used with default search parameters criteria to precisely identify effective and compatible primer sequences. The analytical specificity of primer pairs was preliminarily tested *in silico* by Primer-BLAST [27]. Selected primers were synthesized and HPLC purified by Eurofins Genomics (Eurofins Genomics GmbH, Konstanz, Germany).

A series of optimization experiments were carried out to assess the best-performing concentration for each primer in the range of 250–700 nM and the best-performing annealing in the range of 55–61.4°C by a gradient PCR.

Real-time PCR reactions contained 5 μl quantiFast SYBR® Green qPCR Master mix (Qiagen, Hilden), the proper quantity of forward and reverse primer, 1 μL of DNA template and ultrapure water to the final volume of 10 μL. Amplifications were performed in a RotorGeneQ (Qiagen, Hilden) and consisted of an initial denaturation at 95°C for 2 min followed by 40 cycles of 95°C for 10s, the best performing annealing temperature for 15s, and 72°C for 20s. Fluorescence was measured once per cycle at the end of the extension step and the Cq values were automatically determined by the device.

### Construction of standard curves for quantitative analyses

Absolute quantification of *S*. *pistaciarum* was achieved by comparison with a standard curve. To build it, genomic DNA was serially diluted with sterile water to yield six final concentrations ranging from 10 ng/rxn to 10 fg/rxn and amplified in three technical replicates. The entire experiment was carried out two times independently. In negative control reactions water replaced template DNA. The standard curve was generated by plotting the DNA amounts against the corresponding Cq value. The determination coefficient (R^2^) was calculated and the amplification efficiency (E), which correlates Cq values of both experiments to the amount of target template, was obtained from the slope of the standard curve [28]. The criteria described by Broeders *et al*. [29] about efficiency (90–110%), linearity (R^2^ ≥0.98) and repeatability (relative standard deviation ≤25%) were used to portray the overall performance of the qPCR assay.

The analytical sensitivity of the assay is defined as the lowest concentration of target DNA at which 95% of the positive samples can be detected (limit of detection–LOD). To practically validate the LOD, 8 replicates of DNA at the LOD concentration and at ten-times higher concentration were tested. The whole experiment was repeated three times.

The effect of external DNA or inhibitors potentially contained in plant tissues was tested by building another standard curve from amplification results obtained by spiking the same concentrations of pure pathogen DNA with 50 ng of pure and putatively infection-free *Pistacia vera* DNA (extracted from asymptomatic samples collected in a *S*. *pistaciarum*-free orchard) with the same number of replicates used above.

### Tests for analytical specificity: Exclusivity and inclusivity

The preliminary *in silico* assessment specificity of the of the primers was achieved through Primer-Blast analysis, exploring the NCBI DNA sequence database and excluding the presence of matching sequences in other microorganisms. Thereafter, it was tested by amplification of DNAs extracted from nine isolates of *S*. *pistaciarum* (Table 1) and 13 fungal isolates known to be commonly isolated from *P*. *vera* from different tissues, such as buds, leaves, and fruits. To harmonize the quantity of DNA, it was adjusted to 10 ng/μl for each sample. A melting curve analysis was also carried out to check the uniqueness of the amplicon and the absence of potential primer dimers.

### Validation on naturally infected samples

Validation of the qPCR assay was carried out on a series of 42 DNA samples, both symptomatic and not. Positive and negative controls were always included in the experiments together with a known concentration standard.

Twenty-two symptomatic leaf samples were collected in a symptomatic and unsprayed orchard in Bronte, known to have a disease history. Twenty additional asymptomatic trees were sampled in another orchard putatively *S*. *pistaciarum*-free, located also in the Bronte area after repeated visual inspections confirming the absence of symptoms.

The collected samples were used for both fungal isolation and qPCR validation. Leaves were split into two parts; half was used for the standard isolation procedure (see the procedure described above). From the remaining half, 100 mg of tissue was ground in a mortar with liquid nitrogen and used for DNA extraction. DNA was extracted following the NucleoSpin® Plant II kit manufacturer’s protocol (MACHEREY-NAGEL).

Each sample was run in duplicate so that the obtained value was an average of both technical replicates. The quantity of *S*. *pistaciarum* DNA was extrapolated from the standard curves using Cq values and normalized as described above.

### Data analysis

The threshold value to distinguish positive or negative qPCR results was automatically defined and the standard curves were generated by the Rotor-Gene Q software version 2.1.0.9. Linear regression of the qPCR standard curves was recalculated with GraphPad Prism version 9.0. The qPCR amplification efficiency was estimated from the slopes of the standard curves using the equation

$$E=10^{‐1/\mathrm{slope}‐1}.$$

For absolute quantification, the standard curve generated was used to estimate the target DNA concentrations in the analyzed samples. Further data analyses were done using Microsoft Excel (Microsoft). For each sample the average Cq, the standard deviation, and the coefficient of variation were determined.

## Results

### qPCR tuning

**Fig 1:**
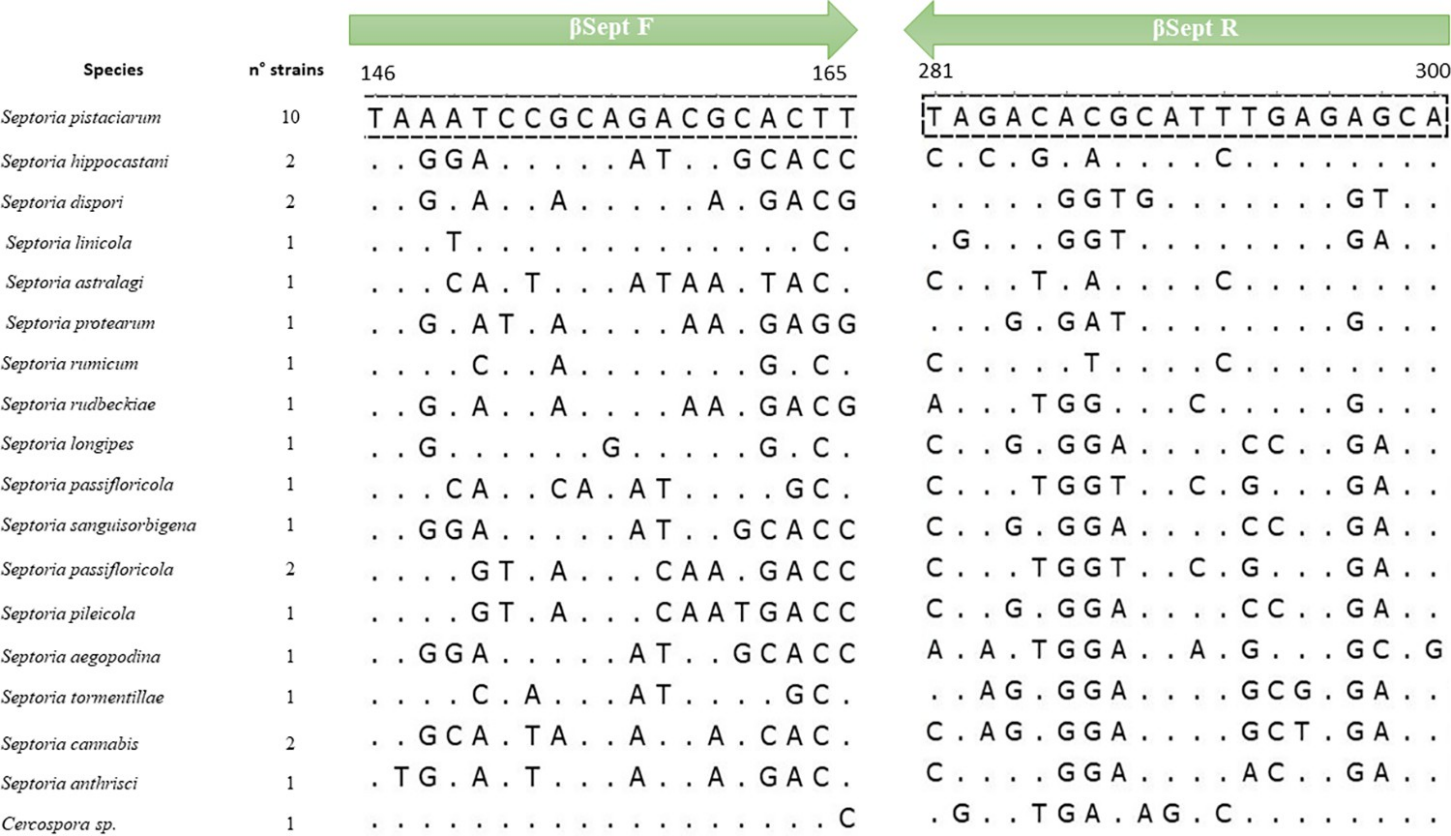
Alignment of primers designed on the β-tubulin gene of *Septoria pistaciarum* and the closest available sequences of species of different genera. The mismatching nucleotides of primers to the sequences of *S*. *pistaciarum* (first line) are reported. The numbers above the alignment refer to positions in *S*. *pistaciarum* strains; The accession numbers of the sequences from NCBI Database are reported below: *Septoria pistaciarum* MZ285918.1, MZ285917.1, MZ285914.1, MZ285913.1, MZ285915.1, KF442739.1, KF442737.1, KF442741.1, KF442740.1, KF442738.1; *Septoria hippocastani* KF252907.1, KF253031.1; *Septoria dispori* MT984358.1, MT984357.1; *Septoria linicola* MZ073925.1; *Septoria astralagi*: KF252821.1; *Septoria protearum*: MT984349.1; *Septoria rumicum*: KF252998.1. *Septoria rudbeckiae*: MN105980.1; *Septoria longipes*: MT984351.1; *Septoria passifloricola*: MK643050.1, MK643054.1, MK643053.1, *Septoria sanguisorbigena*: MT984352.1; *Septoria pileicola*: MT984354.1; *Septoria aegopodina*: KU921453.1; *Septoria tormentillae*: KT861479.1; *Septoria cannabis*: MW556608.1; MW556606.1; *Septoria anthrisci*: KY853401.1; *Cercospora sp*.: KF252781.1.

After the screening for regions suitable for specific primers design in the housekeeping genes, β-tubulin was chosen as the candidate gene for the primer design. The alignment among *S*. *pistaciarum* sequences and 31 sequences belonging to 20 different species, as obtained from NCBI Blastn search, showed stretches of nucleotides highly conserved among *S*. *pistaciarum* isolates and concurrently having substantial differences to the closest relatives (Fig 1). The primer sequences designed in those regions were βSept2 F: 5′- TAAATCCGCAGACGCACTT -3′; βSept2 R: 5′-TGCTCTCARATGCGTGTCTA -3’. The amplicon was estimated to be 152 bp. Based on the analyzed gene sequence, a degenerate nucleotide was included in the reverse primer for the broad detection of the Italian and Turkish isolates.

**Fig 2:**
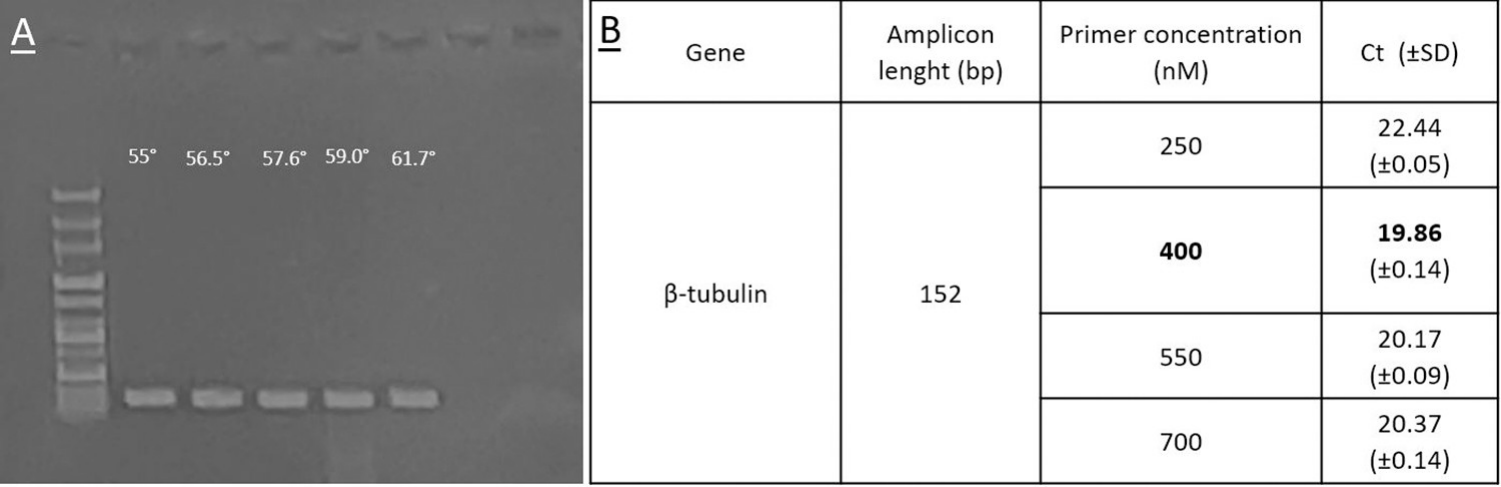
Results of optimization experiments. A) Results of the gradient PCR to select the best annealing temperature from 55 to 61.7°C for the qPCR reaction. B) Optimization of primer concentrations for the qPCR assay for *Septoria pistaciarum*; the best performing concentration for the target gene is highlighted in bold.

The optimization experiments carried out led to the selection of 400nM as the best-performing concentration for the primer pair (Fig 2), and 60° as the best annealing temperature, since no differences were observed between the range of temperatures (55–61.7°C) by a gradient PCR.

### qPCR specificity and standard curves

In Blastn search our primers did not match any DNA sequences among those available in the NCBI database. The following wet lab tests for specificity returned the expected amplicon from the DNAs from nine isolates of *S*. *pistaciarum*, whilst no DNA from non-target fungal genera gave positive amplification (Table 1).

Moreover, the melting curve analysis was performed, and showed a single peak was observed at 83.8°C Tm, confirming the absence of unspecific amplicons or potential primer dimers.

**Fig 3:**
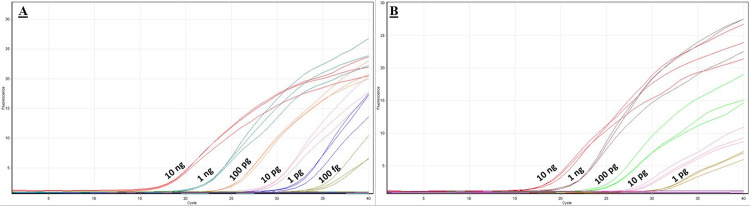
Amplification curves. A) Amplification curves of the qPCR sensitivity test using 10-fold serial dilutions of *Septoria pistaciarum* pure DNA ranging from 10 ng/rxn to 10 fg/rxn. B) Amplification curves of the qPCR sensitivity test using 10-fold dilutions of spiked fungal DNA with *Septoria pistaciarum* with 50 ng of plant DNA.

**Fig 4:**
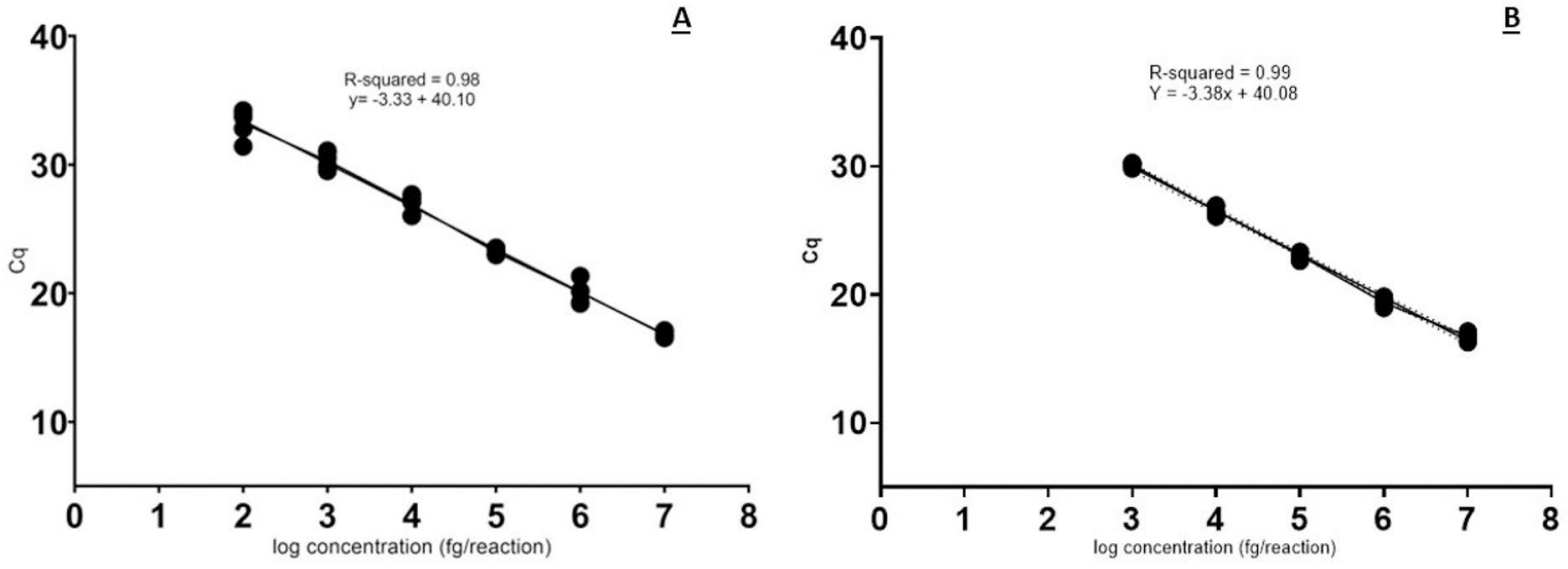
Standard curves. A) Standard curve obtained with 10-fold dilutions of pure DNA extracted from *Septoria pistaciarum* strain S7 (6 replicates) and related statistics. B) Standard curve obtained with 10-fold dilutions of fungal DNA spiked with 50 ng of plant DNA (6 replicates) and related statistics.

The standard curve generated by plotting the six 10-fold dilutions of DNA obtained from the pure fungal culture of strain S7 against the cycle threshold (Ct) of qPCR replicates was linear with a determination coefficient R^2^ of 0.98 and a reaction efficiency of 100% (Figs 3A and 4A). Concerning sensitivity, the lowest DNA concentration returning positive amplifications was 100 fg.

In the assay where the same 10-fold dilutions of pathogen DNA were spiked with DNA obtained from *P*. *vera* (50 ng), the lowest detected concentration of the pathogen was 1 pg, ten times higher than using only pure fungal DNA. The reaction efficiency was 100% and the coefficient of determination (R^2^) was 0.99 (Figs 3B and 4B).

The repeatability of the assay was evaluated in three independent experiments where 8 replicates of fungal DNA diluted to the LOD (1 pg) and ten-time higher concentration (10 pg) were amplified. All 24 samples at LOD concentration (1 pg) tested positive with an average Ct of 27.86 (± 0.84) in the first experiment, 27.9 (± 0.82) in the second, and 27.78 in the third (Table 2). The overall repeatability (CV) was 2.89%. The amplification of samples at the concentration ten-fold higher than LOD was also positive in all 24 reactions.

**Table 2:** Validation of the limit of detection and repeatability of the qPCR method for *Septoria pistaciarum* detection.

Experiments	Run1 (Cq ±SD)	Run2 (Cq ±SD)	Run3 (Cq ±SD)	Repeatibility (%CV)
10 pg	24.40 ±0.36	24.5 ±0.33	24.31 ±0.48	1.62
1 pg	27.86 ±0.84	27.9 ±0.82	27.78 ± 0.74	2.89

We present amplification results of the DNA dilution successfully tested as the LOD (1 pg/rxn) and the 10-fold higher concentration (10 pg/rxn). Each result represents the average from eight technical replicates of strain S7 gDNA spiked with 50 ng DNA of *Pistacia vera*.

### Validation on naturally infected samples

To test the efficiency of the assay in detecting natural infections of *S*. *pistaciarum*, leaves were sampled in two different orchards and used for DNA extraction and isolation. Among the 22 symptomatic leaf samples, all tested positive to qPCR with Cq ranging from 21.99 (± 0.11) to 28.47 (± 0.48). The pathogen was consistently isolated by traditional cultural methods, with isolation frequency from 30 to 100%, as shown in Table 3. When analyzing the 20 asymptomatic leaves, they all resulted negative for both isolation and qPCR.

**Table 3:** Results of qPCR assay and fungal isolations from pistachio samples symptomatic and not.

Sample code	Plant tissue	Presence of symptoms	Isolation	qPCR Result	Cq value (mean ± SD)	*S*. *pistaciarum* DNA (pg/rxn)
P1	leaf	yes	80%	+	24.49 (± 0.61)	37.55
P2	leaf	yes	70%	+	25.01 (± 0.02)	25.27
P3	leaf	yes	60%	+	21.99 (± 0.11)	203.26
P4	leaf	yes	80%	+	23.39 (± 0.08)	77.02
P5	leaf	yes	80%	+	23.41 (± 0.23)	76.51
P6	leaf	yes	70%	+	23.79 (± 0.05)	58.63
P7	leaf	yes	40%	+	25.25 (± 0.08)	21.40
P8	leaf	yes	80%	+	26.30 (± 0.47)	10.59
P9	leaf	yes	80%	+	24.19 (± 0.37)	45.28
P10	leaf	yes	60%	+	24.86 (± 0.59)	29.17
P11	leaf	yes	100%	+	23.36 (± 0.01)	77.2
P12	leaf	yes	50%	+	26.53 (± 0.37)	8.98
P13	leaf	yes	60%	+	26.75 (± 0.05)	7.58
P14	leaf	yes	80%	+	28.47 (±0.48)	2.37
P15	leaf	yes	50%	+	25.12 (±0.16)	21.57
P16	leaf	yes	80%	+	28.40 (±0.03)	2.41
P17	leaf	yes	60%	+	26.26 (±0.01)	10.75
P18	leaf	yes	80%	+	25.79 (±0.16)	14.80
P19	leaf	yes	70%	+	26.07 (±0.83)	13.11
P20	leaf	yes	30%	+	25.86 (±0.07)	13.99
P21	leaf	yes	30%	+	25.37 (± 0.17)	19.63
P22	leaf	yes	60%	+	27.57 (± 0.05)	4.32
AS1	leaf	no	-	-	-	-
AS2	leaf	no	-	-	33.01 (± 0.03)	-
AS3	leaf	no	-	-	-	-
AS4	leaf	no	-	-	31.14 (± 0.08)	-
AS5	leaf	no	-	-	-	-
AS6	leaf	no	-	-	-	-
AS7	leaf	no	-	-	-	-
AS8	leaf	no	-	-	-	-
AS9	leaf	no	-	-	31.33 (± 0.09)	-
AS10	leaf	no	-	-	-	-
AS11	leaf	no	-	-	-	-
AS12	leaf	no	-	-	-	-
AS13	leaf	no	-	-	-	-
AS14	leaf	no	-	-	-	-
AS15	leaf	no	-	-	-	-
AS16	leaf	no	-	-	-	-
AS17	leaf	no	-	-	-	-
AS18	leaf	no	-	-	-	-
AS19	leaf	no	-	-	-	-
AS20	leaf	no	-	-	-	-

P1 to P22 refer to samples collected from a naturally infected field, instead AS1 to AS20 refer to samples collected from a *Septoria* free field.

## Discussion

Septoria leaf spot is considered the most widespread leaf disease of pistachio around the world. The taxonomic re-classification conducted by Crous *et al*. [15] helped to elucidate the classification of this group of pathogens, now accommodated within three genera, including *Cylindroseptoria*, *Pseudocercospora* and *Septoria s*.*str*. In Sicily, the isolates recently collected and characterized by Gusella *et al*., [16] clustered within the group of *S*. *pistaciarum*, confirming the first description made by Caracciolo [14]. These results led to the identification of *S*. *pistaciarum* as the causal agent of the pistachio leaf spot in Italy [16]. Serious damages are inflicted by *S*. *pistaciarum* on pistachio; under severe attacks, trees defoliate prematurely reducing the amount of carbohydrates produced and stored, ultimately decreasing tree vigor [30]. Moreover, the chemical control of the disease is problematic due to the growing concern about the use of pesticides for the risks to human and environmental health, and the increasingly stringent legislation.

Currently, the detection of *S*. *pistaciarum* relies on isolation techniques. These require significant amounts of labor, and time for completion and when the pathogen titer is low and unevenly distributed in the infected plant, isolation can be challenging. *S*. *pistaciarum* is characterized by a very slow growth on cultural media that makes traditional isolation even more challenging because eventual endophytes from cultured leaf tissues often grow quicker than *S*. *pistaciarum* colonies. Also, as for several species within *Septoria* or associated genera, a reliable identification requires the sequencing of at least two housekeeping genes, e.g. Elongation factor alpha and β-tubulin, in addition to the morphological observations [31].

In this study, we developed a species-specific qPCR assay aiming to improve the detection in pistachio tissues. Since the accumulation rate of mutations in the β-tubulin gene during evolutionary times makes it both variable between fungal species and stable within the single species, it has proven to be a region of choice for the design of highly specific primers [32, 23].

The assay proposed in the present study was tested for specificity and sensitivity. The results showed that βSept2F/βSept2R primers amplified DNA extracted from all *S*. *pistaciarum* isolates tested. No amplification was obtained with the other fungal isolates known to be commonly isolated from *P*. *vera*, indicating that other microorganisms on/into the leaf surface will not interfere with the test.

Regarding sensitivity, a clear amplification product was detected down to 100 fg/rxn with pure fungal DNA. This result is better than those obtained by other authors from the same genus even though using a simple Syber green dye and not TaqMan probe technologies. Indeed, Lin *et al*. [33] reported a sensitivity threshold of 10 pg of *S*. *glycines* in a qPCR assay targeting the same gene (β-tubulin) gDNA.

It is worth noticing that the addition of pistachio DNA to the reaction mixture, imitating the direct testing of field samples, slightly affected the detection limit of the assay which was ten-time lower. However, the assay proved to be consistent in detecting as little as 1 pg/rxn of pathogen DNA with 100% of efficiency. This limit of detection (LOD) was confirmed and validated, and the assay positively detected 100% of the samples (24 replicates) with pathogen concentration well above the required threshold for a reliable LOD value, i.e. 95% [28].

The ability of the qPCR assays to detect the DNA of the pathogen in naturally infected plant tissue was also tested. *S*. *pistaciarum* DNA was detected in all the symptomatic leaves. and all samples from the Septoria-free orchard tested negative proving a correct performance in terms of diagnostic specificity.

This qPCR assay proved to be a sensitive, specific, and rapid molecular-based tool to accurately diagnose and quantify the levels of colonization of *S*. *pistaciarum* tissues. Given that little is known about the infection and colonization behaviors of the pathogen on pistachio, it will be very useful to model and predict disease development. As demonstrated in Greece, Italy and Spain, the optimum temperature for *S*. *pistaciarum* ranges from 18 to 25°C, with the first symptoms and signs visible only around the second half of May [11, 16, 30]. The possibility to apply the qPCR assay during the dormant season will allow for the precise detection of the pathogen in the field during latency and prediction of the disease progression.

In addition, the qPCR assay could help to elucidate some cryptic stages of the life cycle of this pathogen. Recent studies conducted around the world on pistachio leaf spot disease describe the life cycle of the pathogen from the late spring when the symptoms are visible and when it produces pycnidia and then asexual spores [11, 16, 30].

Although Gusella *et al*. [16] according to the literature information described a hypothetical life cycle, no quantitative and reliable information is yet available for the other stages (spermatial and sexual). The qPCR assay developed in this study for *S*. *pistaciarum* could also be applied to describe the accumulation of this pathogen in environmental samples such as leaf debris in the orchard, rainwater, air, and in other tissues of the host including buds and woody tissues. In California, qPCR assays were developed for relevant canker pathogens of stone and nut crops, revealing how this molecular tool is important to understand the dynamics of the pathogen’s population in the orchard [25, 34–36].

To the best of our knowledge, this qPCR assay targeting β-tubulin gene is the first able to quantify *S*. *pistaciarum*. The data on specificity, sensitivity, repeatability, and tolerance to inhibitors as well as the preliminary validation on field samples, demonstrated the usefulness of the assay.

## Supporting information

S1 Raw image Results of the gradient PCR to select the best annealing temperature from 55 to 61.7°C for the qPCR reaction. Related to Fig 2A.
(TIF)

S2 Raw image Results of the gradient PCR to select the best annealing temperature from 55 to 61.7°C for the qPCR reaction. Related to Fig 2A.
(TIF)

S1 Table Accession numbers of the sequences from NCBI Database used for the primers design in this study. (DOCX)

S2 Table Mean Cq values and standard deviations from the sensitivity test using 10-fold serial dilutions of pure *Septoria pistaciarum* DNA ranging from 10 ng/rxn to 10 fg/rxn and spiked *Septoria pistaciarum* fungal DNA with 50 ng of *Pistacia vera* DNA. (DOCX)
