# Supplementary figures and images for: A quantitative PCR assay for the detection and quantification of Septoria pistaciarum, the causal agent of pistachio leaf spot in Italy
Source: PLoS One. 2023 May 19;18(5):e0286130. doi: 10.1371/journal.pone.0286130 (PMC10198544; doi:10.1371/journal.pone.0286130)

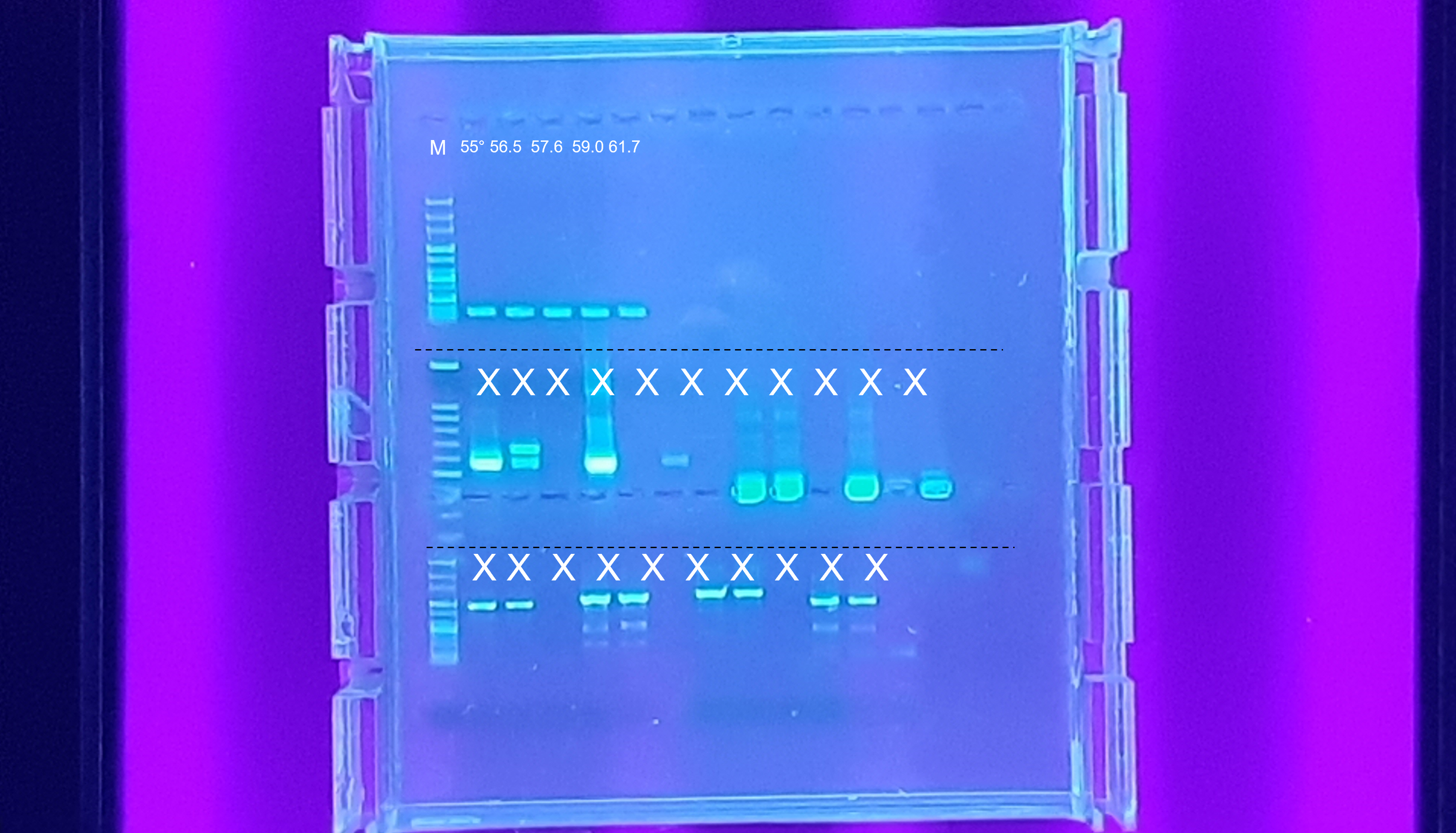

Supplement: S1 Raw image — Related to Fig 2A. (TIF) [file pone.0286130.s001.tif]

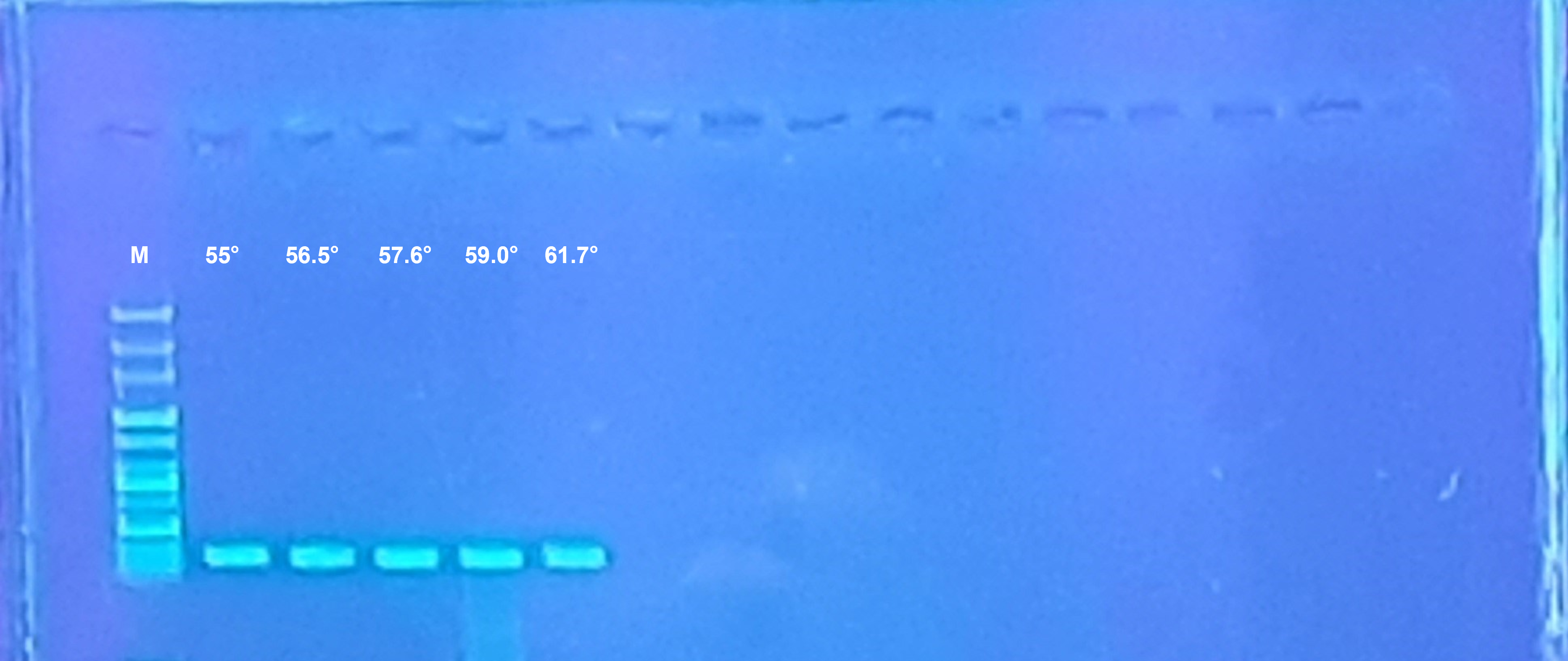

Supplement: S2 Raw image — Related to Fig 2A. (TIF) [file pone.0286130.s002.tif]
