# Supplementary material for: A quantitative PCR assay for the detection and quantification of Septoria pistaciarum, the causal agent of pistachio leaf spot in Italy
Source: PLoS One. 2023 May 19;18(5):e0286130. doi: 10.1371/journal.pone.0286130 (PMC10198544; doi:10.1371/journal.pone.0286130)
Supplement: S1 Table — (DOCX) [file pone.0286130.s003.docx]

**S1. Table. Accession numbers of the sequences from NCBI Database used for the primers design in this study.**

| Species | Isolate | Accession numbers |
| --- | --- | --- |
| *Septoria pistaciarum* | S14 | MZ285918.1 |
|  | SE | MZ285917.1, |
|  | CBS146142 | MZ285914.1, |
|  | CBS146141 | MZ285913.1 |
|  | CBS146143 | MZ285915.1 |
|  | CPC 23115 | KF442739.1 |
|  | CPC 23116 | KF442737.1 |
|  | CBS 135839 | KF442741.1 |
|  | CBS 135838 | KF442740.1 |
|  | CPC 23114 | KF442738.1 |
| *Septoria hippocastani* | CBS 411.61 | KF252907.1 |
|  | MP11 | KF253031.1 |
| *Septoria dispori,* | GUCC 2164.4 | MT984358.1 |
|  | GUCC 2164.3 | MT984357.1 |
| *Septoria linicola* | CBS 316.37 | MZ073925.1 |
| *Septoria astralagi:* | CBS 109117 | KF252821.1 |
| *Septoria protearum:* | GUCC 2127.3 | MT984349.1 |
| *Septoria rumicum:* | CBS 503.76 | KF252998.1 |
| *Septoria rudbeckiae* | RHS454672 | MN105980.1 |
| *Septoria longipes:* | GUCC 2131.1 | MT984351.1 |
| *Septoria passifloricola:* | PLS-S | MK643050.1 |
|  | PLS-R3 | MK643054.1 |
|  | PLS-R2 | MK643053.1 |
| *Septoria sanguisorbigena* | GUCC 2131.2 | *MT984352.1* |
| *Septoria pileicola* | GUCC 2131.4 | MT984354.1 |
| *Septoria aegopodina:* | SAUCC150811 | KU921453.1 |
| *Septoria tormentillae:* | SAUCC130702 | KT861479.1 |
| *Septoria cannabis* | 17JS002 | MW556608.1 |
|  | 18CL004 | MW556606.1 |
| *Septoria anthrisci:* | TCM-11 | KY853401.1 |
| *Cercospora sp.* | CBS 112737 | KF252781.1 |
