# Supplementary material for: A quantitative PCR assay for the detection and quantification of Septoria pistaciarum, the causal agent of pistachio leaf spot in Italy
Source: PLoS One. 2023 May 19;18(5):e0286130. doi: 10.1371/journal.pone.0286130 (PMC10198544; doi:10.1371/journal.pone.0286130)
Supplement: S2 Table — (DOCX) [file pone.0286130.s004.docx]

**S2 Table. Mean Cq values and standard deviations from the sensitivity test using 10-fold serial dilutions of pure *Septoria pistaciarum* DNA ranging from 10 ng/rxn to 10 fg/rxn**

|  | **Pure *S.pistaciarum* DNA** | | ***S.pistaciarum* DNA**  **+**  ***P. vera* DNA** | |
| --- | --- | --- | --- | --- |
| ***S.pistaciarum* concentration (ng/rxn)** | **Mean Cq** | **SD** | **Mean Cq** | **SD** |
| 1 | 16.84 | 0.19 | 16.74 | 0.36 |
| 0.1 | 20.06 | 0.77 | 19.37 | 0.33 |
| 0.01 | 23.21 | 0.23 | 23.07 | 0.30 |
| 0.001 | 26.92 | 0.71 | 26.54 | 0.37 |
| 0.0001 | 30.32 | 0.65 | 30.11 | 0.20 |
| 0.00001 | 33.26 | 1.01 | - | - |

**and spiked *Septoria pistaciarum* fungal DNA with 50 ng of *Pistacia vera* DNA.**
